# Correlation between color doppler flow pattern and molecular biology in elderly patients with colon cancer

**DOI:** 10.1186/s12876-023-02870-9

**Published:** 2023-07-10

**Authors:** Fei Shao, Xiuxiu Lai, Lulu Tong, Linxiao Li, Da Ye, Linlin Jin, Chunyan Xu

**Affiliations:** 1 Rehabilitation Medcine Center, Department of Geriatric VIP No. 3 (Department of Clinical Psychology), Zhejiang Provincial People’s Hospital, Affiliated People’s Hospital, Hangzhou Medical College, Hangzhou, Zhejiang China; 2 Cardiovascular Center, Department of Geriatric VIP No. 6 (Department of Geriatric Medicine), Zhejiang Provincial People’s Hospital, Affiliated People’s Hospital, Hangzhou Medical College, Hangzhou, Zhejiang China; 3 Rehabilitation Medicine Center, Department of Rehabilitation, Encephalopathy Rehabilitation Ward, Zhejiang Provincial People’s Hospital, Affiliated People’s Hospital, Hangzhou Medical College, Hangzhou, Zhejiang China; 4 Cancer Center, Department of Medical Oncology, Zhejiang Provincial People’s Hospital, Affiliated People’s Hospital, Hangzhou Medical College, Hangzhou, Zhejiang China; 5 Cardiovascular Center, Department of Geriatric VIP No. 5 (Department of Geriatric Medicine), Zhejiang Provincial People’s Hospital, Affiliated People’s Hospital, Hangzhou Medical College, 158 Shangtang Road, Gongshu District, Hangzhou, Zhejiang Province China

**Keywords:** Colon cancer, Elderly patients, CDFI, Blood flow grade, Blood flow distribution type, Cytokines

## Abstract

**Objective:**

To investigate the correlation between the grade and type of color Doppler flow imaging (CDFI) and tumor-related cytokines in elderly patients with colon cancer.

**Methods:**

Seventy-six elderly patients with colorectal cancer admitted to Zhejiang Provincial People’s Hospital from July 2020 to June 2022 were selected. CDFI was used to analyze the blood flow grade and distribution type of tumor tissues, and ELISA was used to detect the levels of tumor-related cytokines in serum. Preoperative clinical data were collected and analyzed, and the correlation between measured cytokine levels and CDFI analysis results was further explored.

**Results:**

CDFI blood flow grade showed significant difference in the different lengths, invasion depths and lymph node metastasis of tumors (all *P* < 0.001). In addition, serum levels of TNF-α, IL-6 and VEGF also showed statistical difference in all above different tumor-related factors (all *P* < 0.001). Further Pearson correlation analysis showed that CDFI blood flow grade and distribution types were both significantly positively correlated with above serum cytokine levels (r > 0, all *P* < 0.001). Kaplan-Meier survival analysis showed that both CDFI blood flow grade and distribution types were poor prognostic factors in elderly patients with colon cancer. Regression analysis showed that serum levels of TNF-α, IL-6 and VEGF were independent risk factors for poor prognosis of colon cancer in elderly patients.

**Conclusion:**

CDFI blood flow grade and tumor tissue distribution have potential significant correlations with tumor-associated cytokines in the serum of colon cancer patients. CDFI blood flow grading technique provides an important imaging method for dynamic observation of angiogenesis and blood flow changes in elderly patients with colon cancer. Abnormal changes in serum levels of tumor-related factors can be used as sensitive indicators to evaluate the therapeutic effect and prognosis of colon cancer.

## Introduction

Colon cancer, as a malignant tumor, is located on the colonic mucosa and occurs at the junction of the sigmoid colon and rectum [1, 2]. Colon cancer usually has no apparent symptoms in the early stages [2]. However, severe symptoms, such as dyspepsia, abdominal distension, and even abdominal pain, mucus, or bloody stools, occur in the middle and late stages, which adversely affect the patient’s living quality [3]. Colon cancer is one of the most common cancers worldwide, with almost 1.36 million new cases worldwide each year [4]. Colon cancer has a high prevalence and ranks third in gastrointestinal tumors [4]. With the improvement of living standards, the incidence of colon cancer has been increasing year by year and most pathogenesis occur in the elderly population [5]. Colon cancer is diagnosed at a median age of 69 years, with almost 70% of cases occurring in individuals over 65 years of age and 40% in individuals > 75 years of age [6]. As the aging population continues to intensify, the prevalence of colon cancer will continue to increase.

Recent studies have confirmed that the rapid growth, invasion, spread and migration of colon cancer are closely related to the angiogenesis and distribution of tumors, and the study of the blood flow supply of tumor tissues has become a recent research hotspot [7]. In addition, immune infiltration of the tumor microenvironment seems to differentially influence colon cancer development. Previous studies have reported a correlation between inflammatory cell patterns and prognosis in colon cancer tumors and have also further elaborated on the association between immune cytokines and colon cancer development [7, 8]. Moreover, several tumor-associated cytokines are also related to the development and progression of colon cancer [7]. In essence, this work identifies that analyzing the composition of immune infiltrates improves the accuracy of prognostic information and predictability of response to treatment. Cancer-related inflammatory responses as well as blood flow distribution in tumor tissue have emerged as critical elements to control the clinical behavior of colon cancer [8].

Multiple inflammatory markers, such as tumor necrosis factor α (TNF-α), interleukin-6 (IL-6), and vascular endothelial growth factor (VEGF) have demonstrated prognostic value in patients with colon cancer [9, 10]. In addition, VEGF is also closely related to the blood flow distribution of tumor tissues. However, the association of inflammation-related responses in colon cancer with blood flow distribution in tumor tissue remains unknown for frail older populations at higher risk of developing colon cancer. Therefore, in this study, we analyzed the correlation between Doppler blood flow distribution grade and distribution type as well as tumor-related cytokines in elderly patients with colon cancer in order to evaluate the biological behavior and prognosis of colon cancer in the elderly population.

## Materials and methods

### Study subjects

The clinical data and color Doppler ultrasonography characteristics of patients with colon cancer admitted to the Department of Oncology of Zhejiang Provincial People’s Hospital from July 2020 to June 2022 were retrospectively analyzed.

Inclusion criteria: (1) Radical surgery patients; (2) Pathological diagnosis of colon cancer patients; (3) Patients aged 68 years and above; (4) Patients with complete follow-up data.

Exclusion criteria: (1) Patients who died of non-colorectal cancer during follow-up; (2) Patients with other malignant tumors; (3) Patients who died of serious complications during perioperative period.

According to the inclusion and exclusion criteria, 76 elderly patients with colon cancer were finally included. There were 44 males and 32 females, aged 65 ~ 81 years, with mean one of 70.0 ± 3.7 years. Informed consent was obtained from all patients in this study.

All clinical sample collections were conducted according to the Declaration of Helsinki principles. All recruited patients agreed to conduct the study and this study were approved by the ethical committee of Zhejiang Provincial People’s Hospital with the approval number of QT2023187.

### Instruments and methods

#### Instrument

GE Voluson E10 color Doppler ultrasonic diagnostic apparatus equipped with wide frequency convex array probes of 3.5 and 7 C, with detection frequency of 2.0–10.0 MHz, and equipped with real-time contrast matching imaging technology and curve analysis software. Single molecule phospholipid microbubble shell and sulfur hexafluoride gaseous phase contrast agent were selected. At the time of use, shake well after dissolving with normal saline, then give 2.4 mL contrast agent to the patient to be examined by rapid injection through peripheral vein, and then immediately inject 5 mL normal saline.

#### Doppler ultrasound

Ultrasonography was performed using a color Doppler ultrasound diagnostic apparatus. The contrast agent was dissolved in saline and shaken evenly, followed by 2.4 mL of the patient to be examined by rapid injection into a peripheral vein, followed immediately by 5 mL of saline. All patients were given only a semi-liquid light diet two days before the examination and fasted 8 h before the examination. Appropriate probe frequency was set, and the patients were placed in supine position. Multi-angle exploration was performed on multiple key sites, such as liver, kidney, spleen, and lymph nodes by two-dimensional imaging to observe whether there was lesion metastasis and abdominal lymph nodes.

#### Blood flow grade and distribution

All examinations were performed by the same experienced physician. The size, shape, activity and blood flow of the colon were recorded after the mass was found. CDFI was used to grade the blood flow of the tumor according to the Adler classification (grade 0: no blood flow signal; grade 1: 1–2 blood flow displays were observed; grade 2: 3–4 blood flow displays were observed; grade 3: more than 5 blood flow displays were observed). In addition, following the relationship between the distribution characteristics of blood flow signals and tumors, the blood flow distribution was classified (no blood flow: no blood flow signals were detected in and around the tumor; peripheral type: blood flow was located at the tumor margin and ran along the tumor margin; penetrating type: blood flow ran from the tumor exterior to the center; internal type: blood flow was located inside the tumor and did not reach the tumor margin).

#### Cytokine level detection

All patients underwent fasting blood draws, followed by 30 min of standing, centrifugation, and serum collection. Serum TNF-α, IL-6, and VEGF levels were measured by enzyme-linked immunosorbent assay (ELISA). The kit was purchased from Thermo Fisher Scientific Co., Ltd., and the detection steps were performed according to the kit instructions.

### Statistical methods

Data were analyzed using SPSS 20.0 software. The Kolmogorov-Smirnov test was used for normality testing. Measurement data such as TNF-α, IL-6, and VEGF levels were normally distributed and expressed as x ± s. Independent sample t-test was used for comparison between groups. Enumeration data were analyzed by chi-square test. Pearson correlation was used to analyze the correlation between blood flow grade and distribution and cytokine levels. *P* < 0.05 was considered statistically significant.

## Results

### Comparison of CDFI blood flow grade and tumor-related factors

As shown in Table 1, there were 20 patients with tumor length ≤ 5 cm, of which CDFI blood flow grade 0 accounted for half (10/20 cases); There were 56 patients with tumor length > 5 cm, of which CDFI blood flow was mostly grade 3, accounting for 34% (19/56). The number of cases with CDFI flow grade was statistically different in patients with different tumor lengths (*P* = 0.001). There were 40 patients with tumor invasion depth not invading the serosal layer, of which the majority had CDFI blood flow grade 0, 15 patients in total; there were 36 patients with tumor invasion depth invading the serosal layer, of which the majority had CDFI blood flow grade 3, 17 patients in total. The number of cases with CDFI blood flow grade was statistically different in patients with different depth of tumor invasion (*P* < 0.001). There were 35 patients without tumor lymph node metastasis, of which 14 patients had CDFI blood flow grade 0 and 41 patients had tumor lymph node metastasis, of which 18 patients had CDFI blood flow grade 3. The number of cases with CDFI flow grade was statistically different in patients with or without lymph node metastasis (both *P* < 0.001).

**Table 1:** Comparison of CDFI blood flow grade and tumor-related factors

Content	CDFI Blood Grading				*P* value
	0	1	2	3	
Number	15	19	20	22	
Year	69.5 ± 3.5	71.0 ± 4.6	69.1 ± 2.9	70.3 ± 3.8	
Gender(Male/female)	10/5	12/7	7/13	15/7	
Tumor length					
≤ 5 cm	10	4	3	3	0.001
> 5 cm	5	15	17	19	
Tumor infiltration of serous layer					
-	15	13	7	5	< 0.001
+	0	6	13	17	
Lymphatic metastasis					
-	14	11	6	4	< 0.001
+	1	8	14	18	

### Comparison of cytokine expression levels and tumor-related factors

As shown in Table 2, the expression levels of TNF-α, IL-6 and VEGF were all increased in patients with tumor length > 5 cm compared with patients with tumor length ≤ 5 cm, and the differences were all statistically significant (all *P* < 0.001). The expression levels of TNF-α, IL-6 and VEGF were also increased in patients with tumor invasion depth and serosal layer compared with patients without tumor invasion depth (all *P* < 0.001). Similarly, the expression levels of TNF-α, IL-6, and VEGF were also significantly increased in patients with tumor lymph node metastasis compared with those in patients without tumor lymph node metastasis (all *P* < 0.001).

**Table 2:** Comparison of cytokine expression levels and tumor-related factors

Content	Cytokine expression level		
	TNF-α (ng/mL)	IL-6 (ng/mL)	VEGF (pg/mL)
Tumor length			
≤ 5 cm	197.27 ± 45.20	59.46 ± 9.46	189.82 ± 43.00
> 5 cm	310.33 ± 86.80	77.80 ± 13.40	263.11 ± 64.29
*P* value	< 0.001	< 0.001	< 0.001
Tumor infiltration of serous layer			
-	219.06 ± 54.90	64.71 ± 13.21	211.09 ± 53.54
+	348.92 ± 76.44	82.15 ± 10.64	280.20 ± 63.10
*P* value	< 0.001	< 0.001	< 0.001
Lymphatic metastasis			
-	236.00 ± 71.33	63.68 ± 10.28	191.83 ± 36.45
+	318.63 ± 92.15	80.90 ± 13.54	288.21 ± 55.02
*P* value	< 0.001	< 0.001	< 0.001

### Correlation analysis between CDFI blood flow grade and cytokines

As shown in Table 3, serum TNF-α levels were lowest at 189.60 ± 32.77 ng/L when CDFI blood flow was graded 0, and highest at 322.47 ± 104.49 ng/L when CDFI blood flow was graded 3, respectively. After analysis, CDFI blood flow grade was confirmed to be positively correlated with serum TNF-α levels (r = 0.489, *P* < 0.001). Serum IL-6 levels were lowest at 57.90 ± 8.54 ng/L when CDFI blood flow grade was 0, and highest at 83.37 ± 13.03 ng/L when CDFI blood flow grade was 3, respectively. Moreover, serum VEGF levels were lowest at 185.27 ± 40.70 pg/L when CDFI flow grade 0, and highest at 286.07 ± 57.33 pg/L when CDFI flow grade 3, respectively. After further analysis, it was confirmed that CDFI blood flow grade was positively correlated with serum VFGF levels (r = 0.507, *P* < 0.001) and serum IL-6 levels (r = 0.638, *P* < 0.001).

**Table 3:** Correlation analysis between CDFI blood flow grade and cytokines

Content	CDFI Blood Grading				*r*	*P* value
	0	1	2	3		
TNF-α (ng/L)	189.60 ± 32.77	275.59 ± 81.22	307.47 ± 72.84	322.47 ± 104.49	0.489	< 0.001
IL-6 (ng/L)	57.90 ± 8.54	67.55 ± 10.46	77.98 ± 12.82	83.37 ± 13.03	0.638	< 0.001
VEGF (pg/L)	185.27 ± 40.70	236.12 ± 69.42	248.60 ± 60.87	286.07 ± 57.33	0.507	< 0.001

### Correlation analysis between tumor blood flow distribution types and cytokines

As shown in Table 4, serum TNF-α levels were lowest at 189.60 ± 32.77 ng/L when the blood flow distribution type was no blood flow, and highest at 322.73 ± 106.32 ng/L when the blood flow distribution type was internal. The tumor blood flow distribution type was significantly correlated with serum TNF-α levels (r = 0.451, *P* < 0.001). Serum IL-6 levels were lowest at 57.90 ± 8.54 ng/L when the blood flow distribution type was no blood flow, and highest at 82.78 ± 13.62 ng/L when the blood flow distribution type was internal. There was a significant correlation between tumor blood flow distribution type and serum IL-6 levels (r = 0.550, *P* < 0.001). Serum VEGF levels were lowest at 185.27 ± 40.70 pg/L when the blood flow distribution type was no blood flow and highest at 279.21 ± 51.71 pg/L when the blood flow distribution type was internal. There was a significant correlation between the type of tumor blood flow distribution and serum VEGF levels (r = 0.447, *P* < 0.001).

**Table 4:** Correlation analysis between tumor blood flow distribution types and cytokines

Content	Pattern of blood flow distribution				*r*	*P* value
	Peripheral vascularity	Penetrating vascularity	Central vascularity	Avascularity		
TNF-α	287.65 ± 76.90	305.94 ± 90.14	322.73 ± 106.32	189.60 ± 32.77	0.451	< 0.001
IL-6	71.34 ± 12.87	78.06 ± 13.34	82.78 ± 13.62	57.90 ± 8.54	0.550	< 0.001
VEGF	241.36 ± 73.16	261.89 ± 62.74	279.21 ± 51.71	185.27 ± 40.70	0.447	< 0.001

### Relationship between tumor blood flow distribution and patient prognosis

**Fig. 1:**
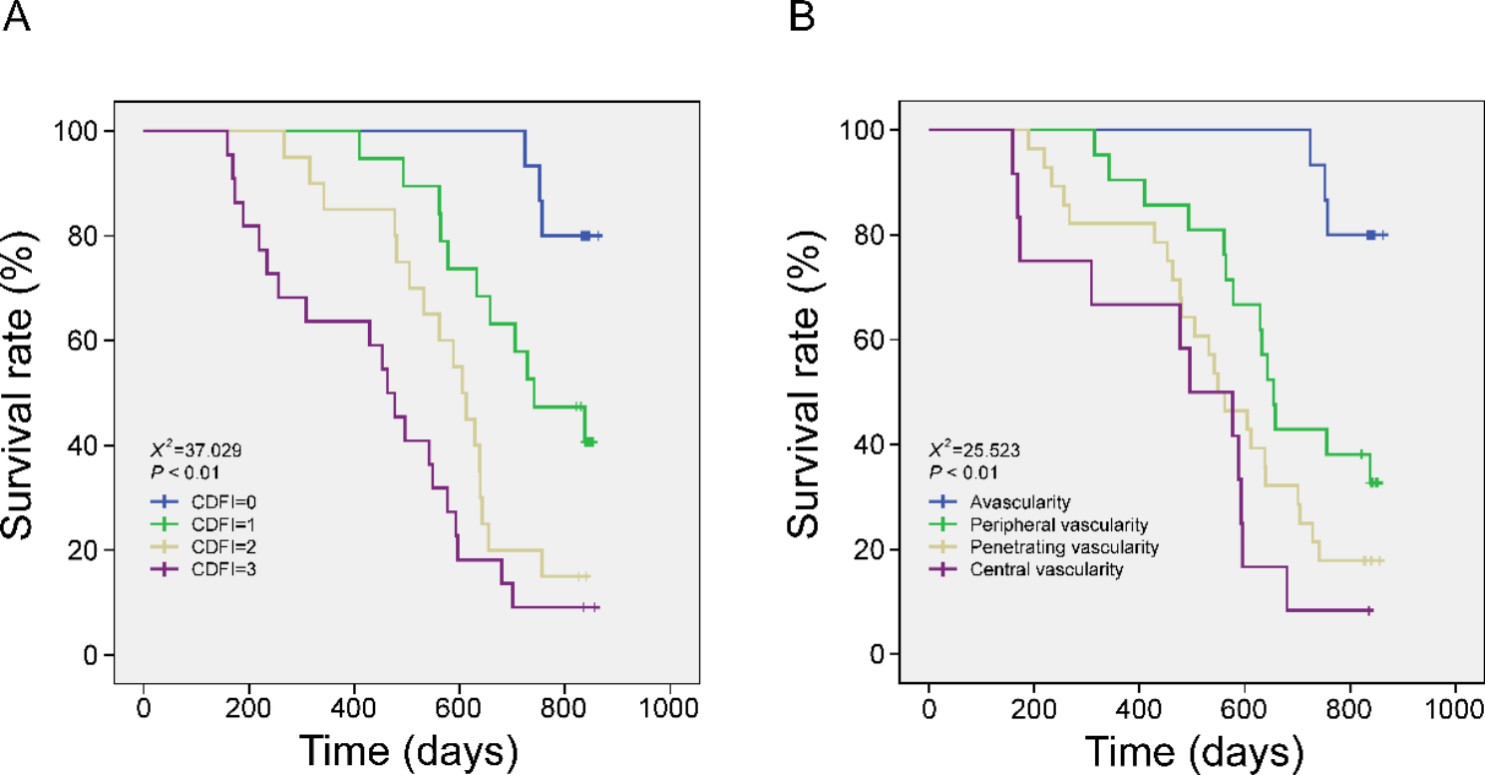
Analysis of overall survival rate in elderly patients with colon cancer

Kaplan-Meier survival analysis was performed to analyze the relationship between survival and CDFI blood flow grade as well as blood flow distribution type in elderly patients with colon cancer. As shown in Fig. 1A, patients with CDFI = 0 or 3 had the highest or lowest survival rate, respectively. Survival distributions were further compared for overall levels of CDFI flow grade by Chi-square test for equivalence, and current results showed that the distribution of different CDFI blood flow grades in the survival rate of elderly patients with colon cancer was statistically different (X2 = 37.029, *P* < 0.001). In addition, patients with no blood flow in the mass had the highest survival rate, while patients with internal blood flow had the lowest survival rate, in the blood flow distribution type. As showed in Fig. 1B, Chi-square test showed that there was a statistically significant difference in the distribution of different blood flow distribution types in the survival rate of elderly patients with colon cancer (X2 = 25.523, *P* < 0.001).

### Relationship between tumor-associated cytokines and patient prognosis

Binary logistic regression was further applied to analyze the influencing factors of survival rate in elderly patients with colon cancer. As the results showed in the Table 5, with the increase of serum TNF-α, IL-6 and VEGF levels, the risk of death in elderly patients with colon cancer was significantly increased (all OR > 1, *P* < 0.001), indicating that TNF-α, IL-6 and VEGF were all independent risk factors for death in elderly patients with colon cancer.

**Table 5:** Relationship between tumor-associated cytokines and patient prognosis

Content	Regression coefficient	SEM	Waldx^2^	Variance	*P* value	OR	95%CI	
							lower limit	Higher limit
TNF-α	0.017	0.005	13.564	1	< 0.001	1.017	1.008	1.026
IL-6	0.082	0.023	13.18	1	< 0.001	1.085	1.038	1.134
VEGF	0.026	0.007	14.962	1	< 0.001	1.026	1.013	1.04

## Discussion

Rapid growth of tumor tissues requires adequate blood supply, while angiogenesis and growth of irregular vascular structures have been identified as significant features of malignant tumors [11]. Tumors can grow in the absence of blood vessels, but reach only 2 mm in diameter [12]. Cancer induces an angiogenic process that promotes the growth of new blood vessels despite their improper construction. Angiogenesis is a very dynamic, multicellular and environmental process. These vessels make local tumor growth and development possible. The phenomenon of increased blood supply early in colon carcinogenesis has been demonstrated in both animal models and human trials [13]. In animal models, increased microvascular blood supply at the precancerous stage has been observed both in azoxymethane (AOM)-treated rats and in mouse models of multiple intestinal tumors of colon tumorigenesis [13, 14]. Vasodilation and increased microvessel density quantified by histological examination have also been detected as the underlying cause of increased blood content in the preadenoma phase [15]. In addition, genetic and environmental factors contributing to local colonic malignant transformation induce more extensive biochemical and molecular changes throughout the colon. The most direct evidence is a significant increase in levels of immune inflammatory factors in vivo.

CDFI blood flow grading technique is widely used and is a non-invasive imaging technique that provides valuable data for evaluating tumor blood flow signals [16, 17]. Previous studies have generally accepted that CDFI has low diagnostic sensitivity and specificity due to its influence by blood flow directions and beam angles. However, with the advancement of transducer technology, high-frequency ultrasound has advantages in macroscopically displaying the generation of tumor vessels because of its physical characteristics, while quantitative or semi-quantitative analysis can also be performed [16]. CDFI can be applied to distinguish various anatomical structures and surrounding tissues, and show the characteristic ultrasonographic findings of diseases such as tumors, cysts, and vascular malformations [18]. Similarly, high-frequency ultrasound can be used to diagnose and localize glomus tumors. In previous studies, tumors < 2 mm in diameter could be identified in high-frequency ultrasound [18]. However, intratumoral vessels in colon cancer differ from normal physiological vessels [18]. Colon cancer has the characteristics of irregular shape, disorganization and uneven distribution of blood vessels in the tumor, and the lumen of blood vessels presents an unnatural state [19]. Spatial distribution and density difference are the pathophysiological basis of CDFI vascular grading technique in detecting colon tumors.

Previous studies have shown that tumor-related factors significantly influence the detection of blood flow at the mass site [20, 21]. Among them, tumor length is one of the most critical factors [22]. This study showed that the majority of colon cancer tumors > 5 cm in size had CDFI flow grade 3 [22]. However, when the tumor length was ≤ 5 cm, the majority of CDFI blood flow grades were grade 0, suggesting that the occurrence and development of colon cancer were significantly associated with the generation of tumor vessels [22]. In colon cancer, the association between blood vessels and clinicopathological factors has been controversia [23]. The studies of Chen et al. and Li et al. as well as our present study did not show any significant correlation [24, 25]. Potential explanations for this discrepancy include the grade of colon cancer development examined, as well as technical limitations imposed on vessel counts at that time. Therefore, more studies are needed to further clarify the level of CDFI blood flow grade in colon tumorigenesis and tumor progression and the significance of its clinical application. Takebayashi et al. additionally evaluated angiogenesis in 166 colorectal cancers and reported a correlation between MVD and depth of invasion and lymph node metastasis [26]. Consistent with the above studies, different CDFI blood flow grades were found to be significantly different in depth of tumor invasion and lymph node metastasis in this study (all *P* < 0.001). The CDFI blood flow grade of colon cancer patients with deep invasion into the serosal layer and lymph node metastasis is generally high, which may be due to the fusion of a large number of new blood vessels in the tumor tissue into larger functional blood vessels after formation, providing nutrients and channels for the growth, invasion and metastasis of colon cancer.

Many previous studies have suggested that angiogenesis, the formation of new blood vessels in the pre-existing vascular bed, plays an important role in tumor growth, maintenance, and metastasis, and that tumor progression to a malignant phenotype depends on the tumor microenvironment [27–29]. Currently, inhibition of tumor angiogenesis is considered a promising strategy for the treatment of cancer. Vascular endothelial growth factor (VEGF) is a potent proangiogenic factor critical for tumor vascular development. When resting endothelial cells are activated, VEGF binds to receptors and signal transduction activates many downstream mediators that allow cells to proliferate, migrate, invade, and eventually differentiate into capillary-like structures. In this study, we found that VEGF levels in serum of colon cancer patients were significantly different in changed tumor lengths, depth of invasion and lymph node metastasis (all *P* < 0.001). When the tumor length was > 5 cm, the depth of invasion invaded the serosal layer, and lymph node metastasis occurred, the VEGF level in the serum was significantly increased, suggesting that VEGF is significantly associated with the formation of the tumor microenvironment. In addition, the results of further regression analysis showed that the risk of death in elderly patients with colon cancer was significantly increased with increasing VEGF levels in serum, suggesting that serum VEGF level is an independent risk factor for poor prognosis in elderly patients with colon cancer.

Previous study have shown that cytokine levels are significantly increased in the serum of colon cancer patients, especially for TNF-α and IL-6 [30]. Therefore, significantly elevated plasma levels of these cytokines may have a good prognostic value in clinical therapy. Therefore, cytokines such as TNF-α are emerging as potential targets for anticancer therapy. For example, TNF-α antagonists have been shown to be well tolerated in patients with solid tumors during clinical trials [31]. Whereas among pro-inflammatory cytokines, recent evidence suggests that IL-6 is a central role linking chronic inflammation to cancer, by driving tumor initiation and subsequent growth and metastasis [32]. In this study, the serum levels of TNF-α and IL-6 in colon cancer patients were significantly different in different tumor lengths, depth of invasion and lymph node metastasis (all *P* < 0.001). Serum levels of TNF-α and IL-6 were significantly increased when tumor length was > 5 cm, depth of invasion into the serosal layer, and lymph node metastasis occurred, consistent with previous findings [33, 34]. Moreover, the results of regression analysis showed that the risk of death in elderly patients with colon cancer increased with the increase of serum TNF-α and IL-6 levels, indicating that TNF-α and IL-6 are independent risk factors for poor prognosis in elderly patients with colon cancer. Although CDFI blood flow grade and different cytokines play an important role in tumor development, the correlation between the two in colon cancer development remains unknown. Pearson correlation analysis showed that CDFI blood flow grade was significantly positively correlated with VEGF levels in the serum of patients (r > 0, *P* < 0.001), that is, the higher the level of VEGF, the higher the CDFI blood flow grade, the richer the tumor blood flow, and the greater the malignancy of colon cancer tumors. Tumor cells not only produce angiogenic factors, but also induce the formation of a variety of immune-inflammatory factors. Serum TNF-α and IL-6 levels also showed a significant positive correlation with CDFI blood flow grade (all r > 0, *P* < 0.001), suggesting that the expression of TNF-α and IL-6 is closely related to the growth, invasion and metastasis of colon cancer tumors. Moreover, further survival analysis showed that the higher the CDFI flow grade, the lower the survival rate of patients, suggesting that patients with high CDFI flow grade had a poor prognosis.

Several previously published studies have shown that the presence of peripheral blood flow in a mass is often associated with benign tumors, whereas the presence of blood flow inside a mass is more likely to be present in malignant tumors [15, 35]. Therefore, evaluation of changes in vascularity profiles may provide valuable diagnostic criteria for the diagnosis of malignancy. Watanabe T et al. [36] used penetrating blood flow as the diagnostic criteria to achieve a diagnostic sensitivity of 68% and a specificity of 95% in malignant lesions of breast cancer. In addition, a study by Xu L et al. showed that poorly differentiated invasive tumors had predominantly internal and penetrating blood flow, while moderately and well differentiated invasive tumors had blood flow mostly located at the periphery of the mass [37]. The tumor vascularity was observed by Doppler ultrasound, and it was found that the number of vessels in malignant tumors was significantly higher than that in benign tumors, and the distribution gradient of vessels was central area > marginal area > surrounding. In this study, the correlation between tumor-associated cytokines and blood flow distribution were further analyzed. It was found that the serum levels of TNF-α, IL-6 and VEGF were significantly higher in colon cancer patients with intratumoral blood flow, while the above cytokine levels were relatively significantly lower in colon cancer patients with intratumoral peripheral blood flow. Current results suggested a significant positive correlation between tumor blood flow distribution and tumor-associated cytokines in elderly colon cancer patients (all r > 0, *P* < 0.001), which further demonstrating that the blood flow distribution has a potential link with the occurrence and development of the tumor. In addition, the results of survival analysis showed that there was also a potential correlation between blood flow distribution and patient survival, and the gradient of vascularity type with poor prognosis was internal type > penetrating type > peripheral type.

Limitations of this study: ① Only patients with colon cancer were studied, cytokines in serum of normal population were not studied and need to be further explored in the future; ② There were few study samples, which could lead to bias in the study conclusions.

In conclusion, CDFI blood flow grade as well as blood flow distribution in tumor tissues have potentially significant correlations with tumor-associated cytokines in the serum of colon cancer patients. CDFI combined with blood flow distribution provides an important imaging method for dynamic observation of angiogenesis and blood flow changes in patients with colon cancer, while abnormal changes in serum tumor-related factor levels are also important for the diagnosis of colon cancer and can be used as a sensitive indicator to evaluate the therapeutic effect and prognosis of colon cancer.

## Data Availability

All data generated or analysed during this study are included in this published article.
